# Analyses of Twelve New Whole Genome Sequences of Cassava Brown Streak Viruses and Ugandan Cassava Brown Streak Viruses from East Africa: Diversity, Supercomputing and Evidence for Further Speciation

**DOI:** 10.1371/journal.pone.0139321

**Published:** 2015-10-06

**Authors:** Joseph Ndunguru, Peter Sseruwagi, Fred Tairo, Francesca Stomeo, Solomon Maina, Appolinaire Djinkeng, Monica Kehoe, Laura M. Boykin

**Affiliations:** 1 Mikocheni Agricultural Research Institute, Sam Nujoma Road, Box 6226, Dar es Salaam, Tanzania; 2 Biosciences eastern and central Africa, The International Livestock Research Institute in Nairobi, Kenya (the BecA-ILRI Hub), P.O. Box 30709, Nairobi 00100, Kenya; 3 Crop Protection Branch, Department of Agriculture and Food Western Australia, Bentley Delivery Centre, Perth 6983, Western Australia, Australia; 4 The University of Western Australia, ARC Centre of Excellence in Plant Energy Biology and School of Chemistry and Biochemistry, Crawley, Perth 6009, Western Australia, Australia; Oklahoma State University, UNITED STATES

## Abstract

Cassava brown streak disease is caused by two devastating viruses, *Cassava brown streak virus* (CBSV) and *Ugandan cassava brown streak virus* (UCBSV) which are frequently found infecting cassava, one of sub-Saharan Africa’s most important staple food crops. Each year these viruses cause losses of up to $100 million USD and can leave entire families without their primary food source, for an entire year. Twelve new whole genomes, including seven of CBSV and five of UCBSV were uncovered in this research, doubling the genomic sequences available in the public domain for these viruses. These new sequences disprove the assumption that the viruses are limited by agro-ecological zones, show that current diagnostic primers are insufficient to provide confident diagnosis of these viruses and give rise to the possibility that there may be as many as four distinct species of virus. Utilizing NGS sequencing technologies and proper phylogenetic practices will rapidly increase the solution to sustainable cassava production.

## Introduction

Cassava brown streak disease (CBSD) presents the most formidable threat to cassava (*Manihot esculenta* Crantz) productivity in sub-Saharan Africa. CBSD is caused by two distinct species of single-stranded RNA (ssRNA) viruses, *Cassava brown streak virus* (CBSV) and *Ugandan cassava brown streak virus* (UCBSV), belonging to the genus *Ipomovirus* of the family *Potyviridae* [1–3]. Both species are reported to be weakly transmitted by the whitefly species complex *Bemisia tabaci* Gennadius in the field [4], and by grafting to indicator plants (e.g. *Nicotiana benthamiana*) or mechanically under laboratory conditions [5]. Similarly, propagating infected cassava cuttings spreads the virus in the field. CBSD was known to be endemic in the coastal East Africa, and inland in parts of Malawi until more recently when outbreaks were reported in Uganda, western Tanzania and Kenya, and in other cassava growing areas including Mozambique, Rwanda, Burundi and in isolated parts of the Democratic Republic of Congo [6]. Collectively, cassava brown streak viruses cause economic losses of up to $100 million USD annually [5].

The complete genomes of various CBSV and UCBSV isolates have been sequenced previously and sizes range from 8,900 to 10,818 nt in length [2, 7]. There are sequence differences between CBSV and UCBSV. For example, the genome of UCBSV is larger (9069 nt as for the case of MLB3 isolate) than that of CBSV (8995 nt for isolate TZ: Kor6: 08) and genetic diversity is wider among the isolates of CBSV (79.3–95.5% at nt level) than UCBSV (86.3–99.3%) [8]. Biologically, in controlled infectivity assays, UCBSV isolates induce systemic mosaic symptoms in *Nicotiana benthamiana* without necrotic local lesions, whereas CBSV isolates cause the latter symptoms [3, 8]. In fields, it is not uncommon to find co-infection of CBSV and UCBSV. In addition, highly susceptible cassava cultivars accumulate higher levels of viral RNA of both CBSV and UCBSV [9]. UCBSV causes milder foliar symptoms than CBSV indicating that CBSV is a more aggressive and virulent CBSD viral pathogen for reasons largely unknown to date [3, 9, 10].

Currently, there are only 12 complete genome sequences of CBSV isolates in GenBank. Additional sequencing of CBSV genomes is key to: 1) developing molecular diagnostic tools for early detection of CBSD-associated virus isolates, 2) generating information on genetic variability, 3) understanding the evolutionary forces acting on the virus genes and 4) clarifying the taxonomy of the virus or viruses associated with CBSD. However, progress has been hampered by conventional methods such as Real-Time PCR that may be too specific to a particular species or even strain of a virus [11]. With the advent of next generation sequencing (NGS) high-throughput sequencing platforms, the capability for random metagenomic sequencing of diseased cassava plants to identify putative viruses has recently become possible [11–13]. Until now, this has been limited by the fact that elimination of the host nucleic acids in the system was critical to enhance viral signals for easy detection, resulting in very low titre viruses, such as CBSV, to be missed. NGS and bioinformatics are now a viable option where sequences from genetic material that are present in the sample (including host and any pathogens such as viruses that may be present) can be generated in a non-specific fashion and identification is based on similarity searching against known virus or virus-like sequences already available in GenBank.

Phylogenetic relationships among the available CBSV whole genome sequences have shown a high level of intra-and inter-genus diversity [2, 7]. However, most of the published phylogenetic trees for CBSV and UCBSV have been constructed based on distance-based methods such as neighbor-joining, which only use matrices to estimate the number of changes there are between species, but does not take into account how each position in the alignments is changing [14, 15]. Such trees only present the tree topology and do not consider branch lengths. Branch lengths are nucleotide substitutions per site in the alignment. They are suitable only when a virus is known to evolve at a constant rate. Another extensively used phylogenetic tree construction method, yet also with limitations, is maximum parsimony, which considers only shared derived characters in the alignment and as a result takes the most parsimonious route without considering branch lengths that would provide information on the rate of virus evolution.

The ideal phylogenetic tree construction methods are the maximum-likelihood and Bayesian methods, which are model-based and use statistics that best describe the data. Maximum-likelihood gives the most likely tree and takes into consideration the rate of change in every single sequence in the alignment (among site rate variation) i.e rate of change in the alignment and branch lengths. A Bayesian approach on the other hand provides many trees by sampling of the tree spaces using Markov Chain Monte Carlo sampling [16] and provides the likelihood of the trees in the tree space. In other words they explore a large area of the tree space to find the global optimum. The numbers on the Bayesian tree are posterior probabilities, which is the probability that the tree is correct, assuming that the model is correct. The only limitation to this method is the time required to complete the analysis. For example, generating phylogenetic trees using MrBayes in Geneious ® 8.0.4 Computer Software, Biomatters Ltd with a default chain length of 1,100,000 for 25 whole genome nucleotide sequences of ipomoviruses may take over 6 hrs to complete using conventional computers.

In this study, we have combined NGS data and the southern hemisphere’s most powerful supercomputer, Magnus, to resolve the phylogenetic relationships of 26 whole genomes of CBSV, including 12 new isolates from Tanzania, Serengeti (in the Lake Zone of Tanzania), Tanga (in the East Coast Zone), Nyasa (in the Southern Zone) and the Mafia district on the island off the Tanzanian mainland. The analysis was performed on 384 cores of the Supercomputer Magnus and provides greater insight into the genomic diversity of CBSV and UCBSV present in sub-Saharan Africa than ever before. The addition of supercomputing applications has allowed for exciting advances in phylogenetic and species delimitation analyses that give us greater confidence in detailing, for the first time, the presence of further closely related species of viruses in the complex mix that makes up CBSD.

## Materials and Methods

### Field sample collection and CBSD symptom assessment

**Fig 1:**
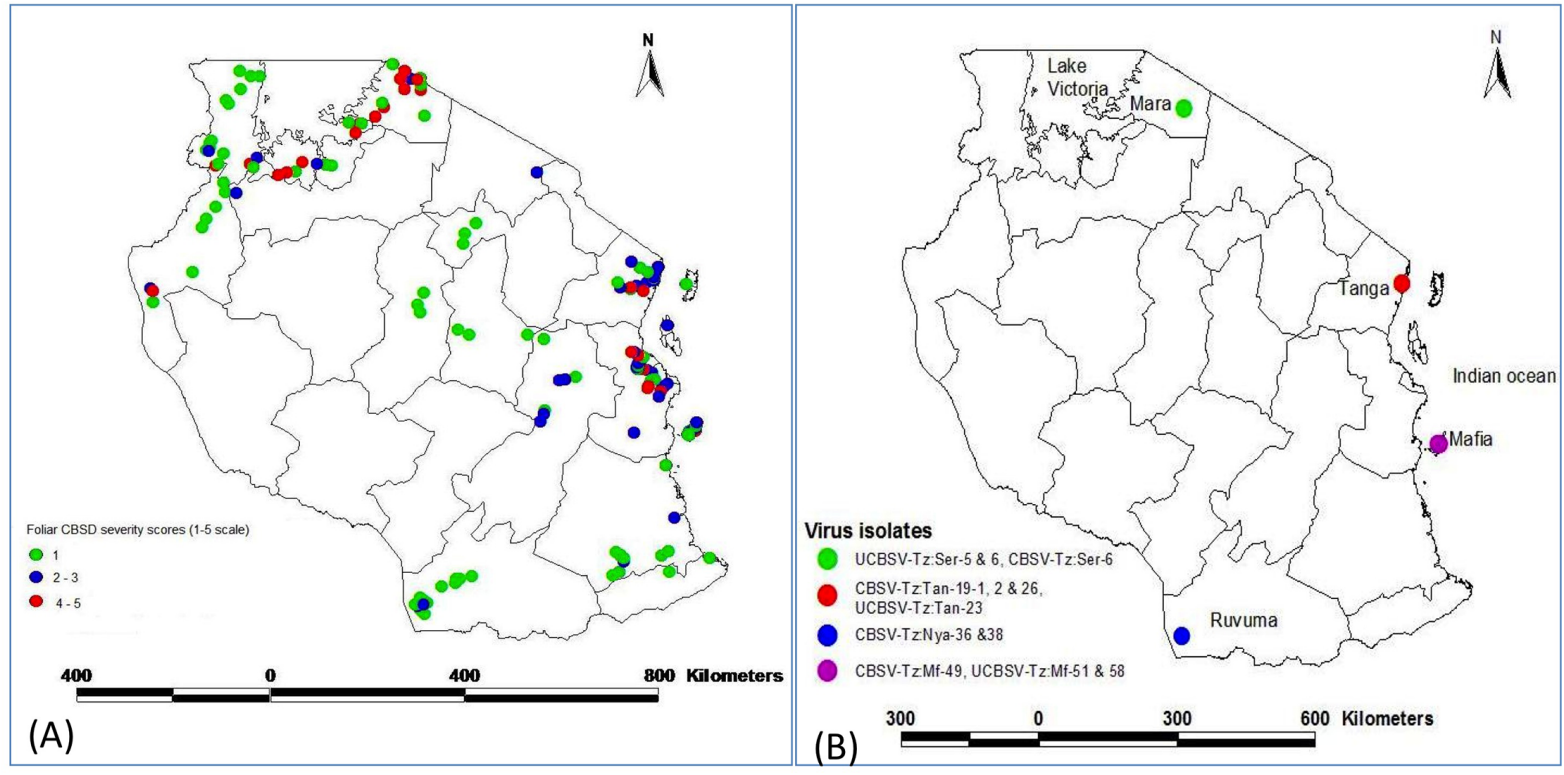
Maps of Tanzania showing the distributions of samples and sequences used in this study. a) Foliar Cassava Brown Streak Disease (CBSD) severity score 1–5. Leaf symptom severity was scored on 3-month-old plants using a five point scale where 1 = no visible CBSD symptoms, 2 = mild foliar symptoms on some leaves, 3 = pronounced foliar symptoms but no die-back, 4 = pronounced foliar symptoms which might include slight die back of terminal branches, and 5 = severe foliar symptoms and plant die-back [17, 18]. b) Geographical distribution of new cassava brown streak virus isolates, CBSVs and UCBSVs used in the present study. Locational names abbreviation in virus isolate names are: Nya = Nyasa; MAF = Mafia; Tan = Tanga, and Ser = Serengeti.

Cassava fields (3–6 months old) in the major cassava growing zones of Tanzania (Coast, Lake, Southern, Zanzibar and Mafia Islands) were inspected for CBSD symptoms (Fig 1). A total of 470 leaf samples were collected from symptomatic cassava plants (displayed in their leaves and/or roots) and transported to the Mikocheni Agricultural Research Institute for further analysis. Smallholder farmers in Tanzania allowed us access to their farms and field studies did not involve endangered or protected species. Leaf symptom severity was scored on 3-month-old plants using a five point scale where 1 = no visible CBSD symptoms, 2 = mild foliar symptoms on some leaves, 3 = pronounced foliar symptoms but no die-back, 4 = pronounced foliar symptoms which might include slight dieback of terminal branches, and 5 = severe foliar symptoms and plant die-back [17, 18]. Root symptoms were recorded about 18 months after planting by horizontally cutting the tubers every 1–2 cm.

### RNA extraction

RNA was extracted from approximately 100mg of cassava leaf using the CTAB (cetyltrimethyl ammonium bromide) [19, 20]. The leaves were ground in a mortar containing 1 ml extraction buffer (2.0% (w/v) CTAB, 2.0 M NaCl, 2.0% PVP, 0.5M EDTA, 1 M Tris-HCl and 0.2% β-mercaptoethanol (added immediately before use). Then 750 μl of the extract was transferred into a 1.5 ml micro-centrifuge tube and incubated at 65°C for 15 min while shaking vigorously several times. The extract was then mixed with an equal volume (750 μl) of chloroform: isoamyl alcohol (24:1); vortexed briefly and centrifuged (Hettich Centrifugen, D–78532, Germany) at 12,000 rpm for 10 min at 4°C. The top aqueous solution (500 μl) was transferred into new micro-centrifuge tubes to which 0.6 vol (300 μl) cold isopropanol was added. The content was then incubated at -20 for at least 10 min followed by centrifugation (Hettich Centrifugen, D–78532, Germany) at 13,000 rpm for 10 min at 4°C and the supernatant was discarded. The RNA pellet was then washed in 700 ml of 70% ethanol and the tubes vortexed briefly before being incubated at -20°C for at least 10 min. The tubes were then centrifuged for 5 min at 13,000 rpm. The ethanol was then removed and the pellet was air-dried. Finally the dried RNA pellet were re-suspended in 100 μl 1XTE/sterilized double distilled H20 on ice for about 30 min and stored at -20°C before use.

### cDNA library preparation and *Illumina* ^*®*^ sequencing

Total RNA extracts that presented 260/280 and 260/230 purity indices equal to or greater than 2.0 and integral RNA in electrophoresis and Bioanalyzer measurements (RIN>8) were selected. The cDNA libraries were prepared from 1 μg of total RNA using the IlluminaTruSeq Stranded Total RNA Sample Preparation kit with Ribo-Zero^TM^Plant according to the manufacturer’s instructions (Illumina, San Diego, California). Briefly, after rRNA depletion and RNA fragmentation, first and second strand cDNA was synthesized, adapters were ligated to the 5′ and 3′ ends of the fragments and the fragments enriched by PCR. cDNA libraries final size and concentration of each library was estimated using a Bioanalyzer (Agilent, Santa Clara, CA, USA) and the Qubit (Invitrogen, Carlsbad, CA, USA), respectively. Ten nM library pools were prepared by mixing the libraries to achieve an equal molar concentration of each. Libraries were normalized, pooled and sequenced using a 2×300 -cycle PE V3 Illumina kit. Paired end reads were generated using the Illumina MiSeq System at the Biosciences Eastern and Central Africa–International Livestock research Institute (BecA-ILRI) Hub in Nairobi, Kenya.

### De novo Sequence Assembly and mapping

For each sample, reads were first trimmed using CLC Genomics Workbench 6.5 (CLCGW) (CLC Bio) with the quality scores limit set to 0.01, maximum number of ambiguities to two and removing any reads with <30 nucleotides (nt). Contigs were assembled using the *de novo* assembly function of CLCGW with automatic word size, automatic bubble size, minimum contig length 500, mismatch cost two, insertion cost three, deletion cost three, length fraction 0.5 and similarity fraction 0.9. Contigs were sorted by length and the longest subjected to a BLAST search (blastn and blastx) [21]. In addition, reads were also imported into Geneious 6.1.6 [22] (Biomatters) and provided with reference sequences obtained from Genbank (NC012698 for CBSV, GQ329864 for CBSV-T and NC014791 for UCBSV). Mapping was performed with minimum overlap 10%, minimum overlap identity 80%, allow gaps 10% and fine tuning set to iterate up to 10 times. A consensus between the contig of interest from CLCGW and the consensus from mapping in Geneious was created in Geneious by alignment with MAFFT [23]. Open reading frames (ORFs) were predicted and annotations made using Geneious. Finalized sequences were designated as “complete” based on comparison with the reference sequences used in the mapping process, and “coding complete” if some of the 5’ or 3’ UTR was missing but the coding region was intact [12, 24], and entered into GenBank accession number KR108828-KR108839 (Table 1)

**Table 1:** Next generation sequencing data from Cassava Brown Streak Disease symptomatic plants collected in Tanzania.

No. of reads obtained	No. of reads after trimming	Number of contigs produced (CLC)	Sample ID	Accession number	Virus	Average coverage (CLCGW)	Number of reads mapped to contig of interest	Ref seq. used for mapping	Length of consensus sequence from mapping (Geneious)	No. reads mapped to ref. sequence	Average coverage (Geneious)	Final sequence length
2,790,456	2,743,551	224	**Tz:Ser–5**	KR108838	*UCBSV*	39	2,694	NC014791	9,045	2,701	40	9,045
4,988,502	4,902,387	5,761	**Tz:Ser–6**	KR108830	*CBSV*	721	48,557	GQ329864	9,939	49,252	673	8,994
			**Tz:Ser–6**	KR108837	*UCBSV*	613	41,545	NC014791	9,276	41,496	620	9,060
2,735,840	2,690,100	3,820	**Tz:Tan-19-1**	KR108834	*CBSV*	146	8,252	NC012698	9,865	7,350	104	8,945
			**Tz:Tan-19-2**	KR108832	*CBSV*	103	5,469	GQ329864	9,057	9,033	143	8,973
4,978,272	4,900,398	7,411	**Tz:Tan–23**	KR108839	*UCBSV*	702	45,275	NC014791	9,882	47,433	670	9,070
2,732,618	2,732,096	3,558	**Tz:Tan–26**	KR108833	*CBSV*	66	4,028	GQ329864	9,026	4,169	67	8,918
5,719,724	5,582,134	1,846	**Tz:Nya–36**	KR108831	*CBSV*	50	2,027	GQ329864	10,044	5,044	66	8,987
2,560,544	2,518,097	6,022	**Tz:Nya–38**	KR108829	*CBSV*	573	37,313	NC012698	9,736	32,857	543	8,985
2,088,040	2,054,816	2,674	**Tz:Mf–49**	KR108828	*CBSV*	263	17,354	NC012698	8,549	14,186	237	8,996
2,071,164	2,025,314	4,254	**Tz:Maf–51**	KR108836	*UCBSV*	223	12,779	NC014791	9,175	14,289	234	9,066
2,548,594	2,509,206	4,578	**Tz:Maf–58**	KR108835	*UCBSV*	213	14,426	NC014791	9,799	15,386	213	9,056

### Genome alignment

Twelve CBSV (5) and UCBSV (7) whole genomes were downloaded from GenBank and imported into Geneious, and the Mauve plugin was used to align these with the 12 new whole genome sequences. Nucleotide alignments were translated into protein using the MAFFT translate align option in Geneious and then visually inspected.

### Recombination detection

The RDP4 package [25] was used to detect recombination between the 26 whole genome sequences. Default parameters were used for the seven programs implemented within RDP: RDP [26], GENECONV [27], Bootscan [28], MaxChi [29], Chimaera [30], 3Seq [31] and SiScan [32] which included using a Bonferroni corrected *P* value cutoff of 0.05. A recombination pattern was considered if detected by four or more of these programs, and anything less than four programs were not considered a valid recombination event [24, 33, 34].

### Bayesian phylogenetic analyses

**Fig 2:**
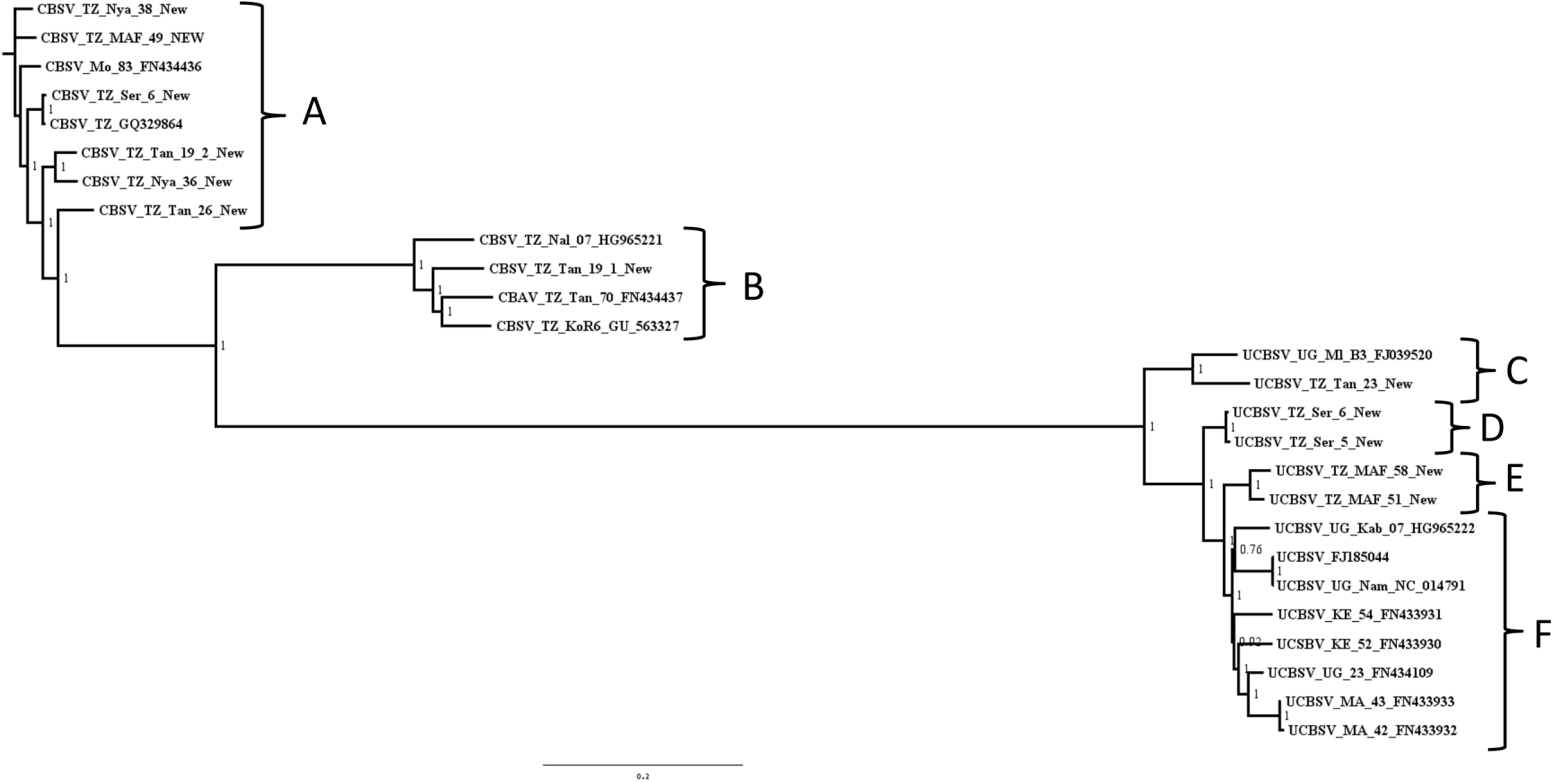
Whole CBSV/UCBSV genomes (nucleotides) were analyses using a Bayesian analyses were conducted using ExaBayes version 1.4.1; [35] and were run in parallel across 384 nodes on the Magnus supercomputer (located at the Pawsey Centre, Western Australia). Analyses were run for 1 million generations with sampling every 500 generations. Convergence was evaluated using Tracer. Each analysis consisted of four independent runs, each utilising four coupled Markov chains.

Bayesian analyses were conducted using ExaBayes version 1.4.1; [35] and were run in parallel across 384 nodes on the Magnus supercomputer (located at the Pawsey Centre, Western Australia). The Magnus supercomputer consists of eight cabinets, each with 48 blades and four nodes per blade. Each node contains two 12-core Intel Xeon E5-2690V3 Halswell processors with 2.6 GHz. Analyses were run for 1 million generations with sampling every 500 generations. Each analysis consisted of four independent runs, each utilising four coupled Markov chains. The run convergence was monitored by finding the plateau in the likelihood scores (standard deviation of split frequencies < 0.0015). Convergence of other parameters was also checked during post-processing and included the commands consense–f ExaBayes_topologies.test.* -n consextractBips-f ExaBayes_topologies.test.* -n bips, credibleSet -f ExaBayes_topologies.test.* -n cred, postProcParam -f ExaBayes_parameters.test.* -n param. The first 25% of each run was discarded as burn-in for the estimation of a majority rule consensus topology and posterior probability for each node. Bayesian run files are available from the authors upon request. Each analysis was repeated three times to ensure consistency in the consensus trees and convergence of parameters. The whole genome nucleotide and amino acid alignments were also analysed for the individual genes using ExaBayes and following the same strategy. The complete genome tree (Fig 2) was used as the reference tree and all other individual gene trees were compared to it. A (+) indicates that the clade contains the same species as Fig 2 and occurs in the same location on the tree. A (-) indicates that the clades differ when compared to Fig 2 in either species delimitation and/or are in a different location on the tree.

### Species delimitation

The tip to root approach of species delimitation [36] was employed on the whole genome nucleotide ExaBayes tree (Fig 2). Starting at the tips of the tree and working towards the root, species delimitation measures were obtained for every clade with a minimum of two taxa and values recorded. The tip to root process is designed to delineate species objectively as the species delimitation measures dictate where to draw the “species” line. An individual without prior knowledge of the current species determination performed the species delimitation. Species delimitation was addressed using the standard Kimura two-parameter (K2P) inter-species distance plus two more stringent measures of taxon distinctiveness, 1) Rosenberg’s reciprocal monophyly, P(AB) [37] and 2) Rodrigo’s P(randomly distinct) [38]. The species delimitation plugin [39] for Geneious [22] was used to calculate Rosenberg’s reciprocal monophyly, P(AB) [37] and Rodrigo’s (P(RD) measures [38].

## Results

### Geographical distribution of CBSV and UCBSV in Tanzania

Based on the analysis of whole genome virus sequences, both CBSV and UCBSV are widely distributed in Tanzania. In coastal areas, CBSV and UCBSV occurred widely. Three CBSV isolates (TZ: Tan-19-1, 19–2 and 26) were identified in the Tanga district from unnamed local CBSD-affected cassava plants. One UCBSV isolate (TZ: Tan–23) was found to occur in the same district. One CBSV isolate (TZ: MAF–49) and two UCBSV isolates (TZ: MAF–51 and 58) were identified from cassava cultivar Kilembe in Mafia district, an island located in the Indian Ocean off the Tanzanian mainland. In the Lake zone in northwestern Tanzania, in Serengeti district of the Mara region, two CBSV isolates (TZ: Ser–5 and 6) were identified from Rumala, a local cassava cultivar. Another isolate UCBSV (TZ: Ser–6) was found in a co-infection with a CBSV isolate (TZ: Ser–6) in the same plant. In southern Tanzania, we found two CBSV isolates (TZ: Nya–36 and 38) from CBSD-affected cassava cultivar ‘Nkangawandu’ in the Nyasa district, on the shore of the Lake Nyasa near Mbamba Bay in the Ruvuma region. These findings represent the first report of CBSD occurrence in the district. The geographical locations from where the CBSV and UCBSV were collected, symptom severity score distribution and the identity of the genetic group of the isolates are shown in Fig 1A and Fig 1B.

### CBSD field symptoms associated with CBSV and UCBSV virus isolates

**Fig 3:**
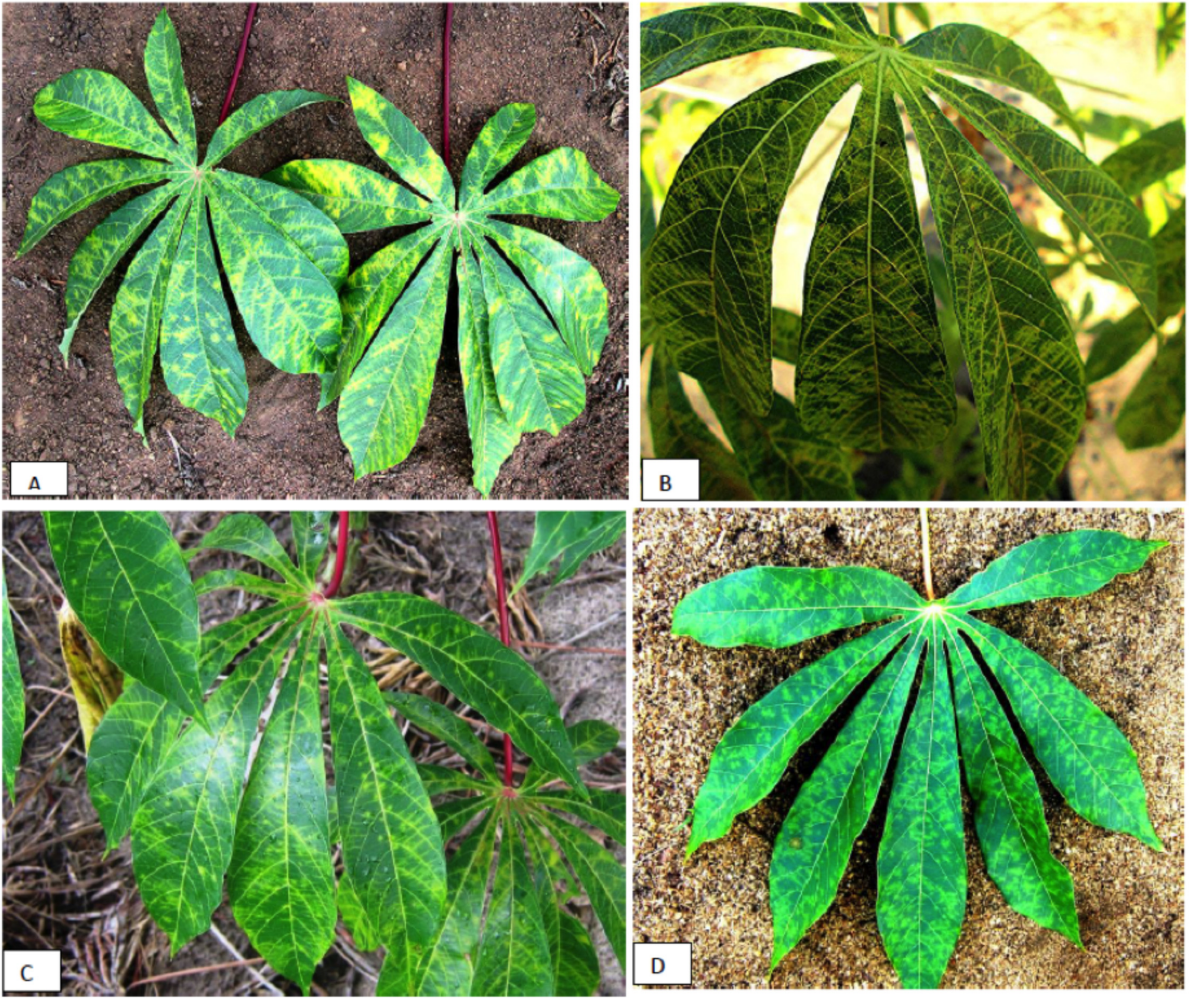
Cassava brown streak disease symptoms seen on infected cassava plants collected from the field; a) Mafia district, feathery patterns with chlorotic blotches along the margins of secondary, tertiary and main veins; b) Tanga district, severe yellow chlorosis on leaves; c) Nyasa district, moderate leaf chlorosis and d) Serengeti district, moderate chlorosis, mottling and chlorotic blotches.

Foliar CBSD symptom types and severity varied from mild to severe on the plants that were surveyed. Field infected cassava plants collected in Mafia district from which the CBSV isolate TZ:MAF–49 and the UCBSV isolates TZ:MAF–51 and 58 were found displayed pronounced varying patterns of chlorosis, which appeared as feathery patterns with chlorotic blotches along the margins of secondary veins, tertiary veins, and main veins (Fig 3A). In addition, some CBSD-affected plants expressed interveinal circular patches of chlorosis. Mean CBSD symptom severity scoring scale was 3±0.45 (SE). Upon uprooting of the cassava roots, no root necrosis symptoms were observed.

In the Tanga district, cassava plants infected by UCBSV isolates TZ:Tan-19-1 and 2, TZ:Tan–26 and UCBSV isolates TZ:Tan–23 displayed severe yellow chlorosis on leaves (Mean symptom severity scoring scale of 4±0.75 (SE) (Fig 3B). Die-back and severe root necrosis was observed on plants that were doubly infected by CBSV isolates TZ:19–1 and 2. In Nyasa district, plants infected by CBSV isolates TZ:Nya–36 and 38 displayed moderate to severe leaf chlorosis (Fig 3C) but showed no root necrosis. The three isolates (UCBSVTZ:Ser–5 and 6 and CBSVTZ:Ser–6) were associated with mild to moderate chlorosis, mottling, and chlorotic blotches (Fig 3D) without root symptoms. The CBSD foliar symptom severity scoring scale averaged 2±0.80 (SE).

### Next generation sequencing

Ten samples were sequenced on an Illumina MiSeq, which produced numbers of raw reads ranging from 2,071,164 to 5,719,724 for each sample. Raw reads can be accessed from the European Nucleotide Archive accession number: PRJEB10634. After trimming for quality using CLCGW, these numbers were reduced to 2,025,314 to 5,582,134 (Table 1). Following *de novo* assembly of the reads for each individual sequence, also using CLCGW, the number of contigs produced ranged from 224 to 7,411. The contig of interest lengths were 5,339 to 9,074 with average coverage of 39 to 721 times and the numbers of reads mapped to each contig were 2,027 to 48,557. After mapping to a reference genome in Geneious, the lengths of the consensus sequences were 8,549 to 10,044 with average coverage of 40 to 673 times with the numbers of reads mapped to the reference sequence ranging from 2,701 to 49,252. Final overall sequence lengths consisted of a consensus between the *de novo* contig of interest and the mapped consensus sequence and were 8,945 to 9,070 nt. Eight of the samples yielded one sequence of interest each and two others yielded two sequences of interest each, with a total of 12 new sequences, five of UCBSV and seven of CBSV. All sequences were submitted to GenBank with the accession numbers KR108828-KR108839. Raw reads forming part of the assembled genomes can be made available to individuals upon request.

### Recombination

**Fig 4:**
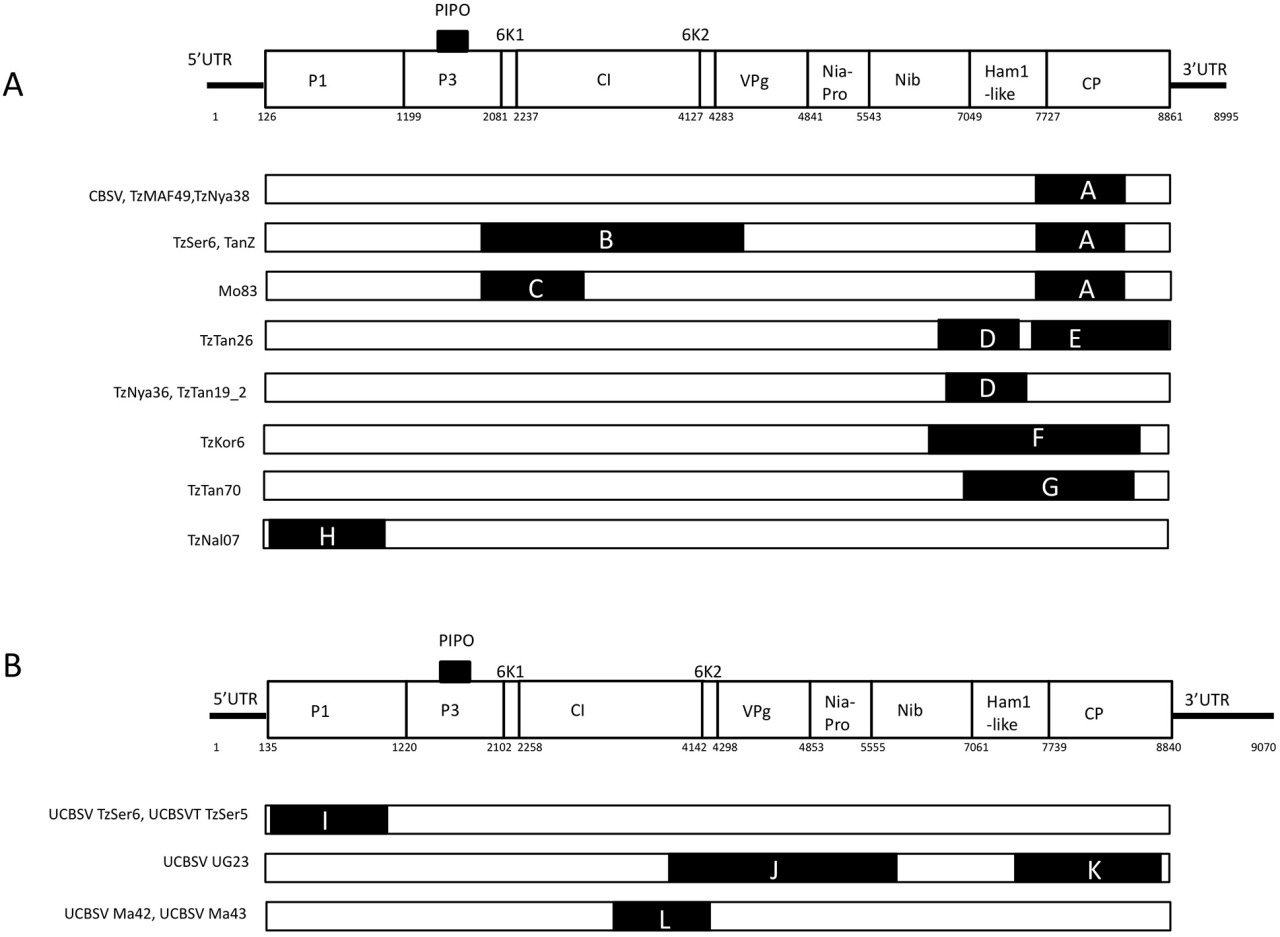
Recombination patterns observed from analysis involving the 12 new sequences along with those retrieved from GenBank; a) Eight firm recombination events identified amongst the new Cassava brown streak virus sequences when compared with all those available on GenBank; and b) Four firm recombination events identified amongst the new Ugandan cassava brown streak virus sequences when compared with all those available on GenBank.

When the complete coding regions of the 12 new sequences, along with those retrieved from GenBank were analyzed, eight firm recombination events were identified amongst the CBSV sequences (Table 2, Fig 4A) and four were identified amongst the UCBSV sequences (Table 3, Fig 4B). For CBSV, isolates CBSV, TzMaf49, TzNya38, TzNya36, TzTan19_2, TzKor6, and TzTan70 all contained just one single recombination event each at the 3’ end of the genome (Ham1-like and CP). TzNal07 also had just one event, although it occurred at the 5’ end in the P1 gene. Isolates Tz-Ser–6, Tan-Z, Mo–83 and Tz-Tan–26 all contained two events each in the CI region as well as the 3’ end of the genome. For UCBSV, isolates UCBSV Tz-Ser–5 and UCBSV Tz-Ser–6 each had one recombination event in the P1 region at the 5’ end of the genome. Isolates UCBSV Ma–42 and UCBSV Ma–43 each contained one event in the CI region in the middle of the genome and UCBSV UG–23 contained two events, one in the area of the CI-6K2-VPg-Nia-Pro and another across the Ham1-like and CP regions. The parental sequences in all CBSV recombination events were other CBSV sequences, and the parental sequences for all UCBSV events were also other UCBSV sequences.

**Table 2:** Recombination events in the coding regions of *Cassava brown streak virus* (CBSV) genomes when analyzed with CBSV and *Ugandan cassava brown streak virus* genomes collected in Tanzania.

Event	Recombinant sequences	Programs detected by	Start position in alignment	Gene region	Parental sequence	P-value
A	CBSV, TzMaf49, TzNya38, TzSer6, TanZ, Mo83	R,G,B,M,C	6621–8006	Ham1-like,CP	TzNya36 x unknown	1.663x10^-07^
B	TzSer6, TanZ	R,B,M,C, Si, 2	2013	P3,6K1,CI,6K2,VPg	TzNya38 x TzTan19_2	2.893x10^-10^
C	Mo83	R,B,M,C,S,3	2013–2591	P3,6K1,CI	TzMaf49 x TzTanZ	2.670x10^-08^
D	TzTan26, TzNya36, TzTan19_2	R,G,B,M,C,S	6469–6663	Nib,Ham1-like	TzNya38 x TzNal07	7.545x10^-21^
E	TzTan36	R, B,M,C	7854–7991	Ham1-like,CP	TzMaf49 x TzTan19_1	1.098x10^-08^
F	TzKor6	R,B,M,C,S	6136	Nib,Ham1-like,CP	TzTan19_1 x TzNal07	1.823x10^-08^
G	TzTan70	R,G,B,M,C,S	6808	Nib,Ham1-like,CP	TzTan19_1 x unknown	2.982x10^-15^
H	TzNal07	R,G,B,M,C,S	47	P1	Kor6 x unknown	6.819x10^-21^

**Table 3:** Recombination events in the coding regions of *Ugandan cassava brown streak virus* (UCBSV) genomes when analyzed with UCBSV and *Cassava brown streak virus* genomes.

Event	Virus	Recombinant sequences	Programs detected by	Start position in alignment	Gene region	Parental sequence	P-value
I	UCBSV	UCBSV TzSer6, UCBSV TzSer5	R,G,B,M,C,S,3	0–227	P1	UCBSV Maf51 x UCBSV UGM1B3	5.474x10^-24^
J	UCBSV	UCBSV Ug23	R,G,B,M,C,S,3	3826–4577	CI,6K2,VPg,Nia-Pro,Nib	UCBSV KE52 x UCBSV KE54	6.859x10^-35^
K	UCBSV	UCBSV Ug23	R,G,B,M,C,S,3	7435–7560	Ham1-like,CP	UCBSV Ke52 x UCBSV UGKab_07	2.813x10^-18^
L	UCBSV	UCBSV Ma42, UCBSV Ma43	R,G,B,M,C,S	3814–3980	CI, 6K2	UCBSV UG23 x unknown	1.441x10^-04^

### Bayesian phylogenetic analyses

**Fig 5:**
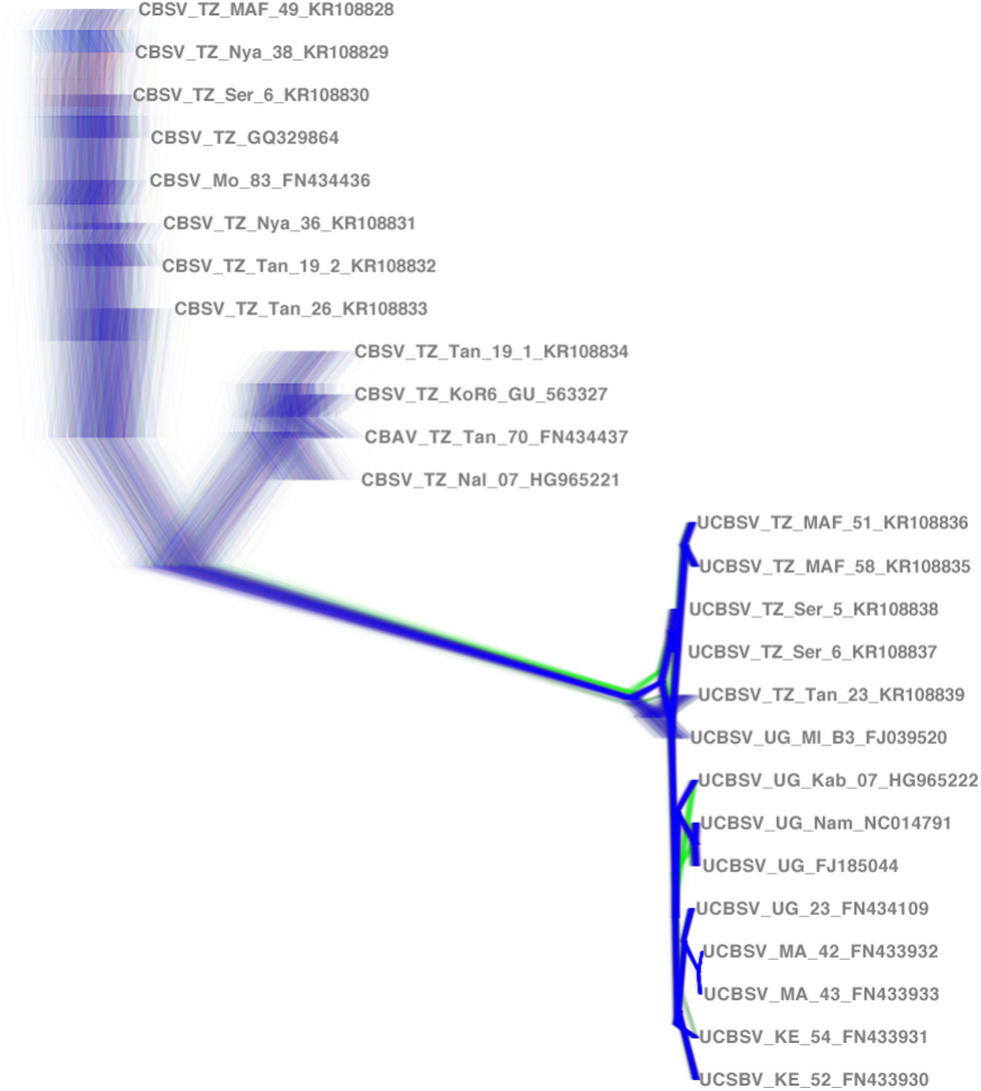
DensiTree [40] is a visualization tool that displays all trees encountered as the Markov Chains samples the large tree space. Two-thousand trees are depicted in the Densitree and there is clearly more uncertainty in the CBSV relationships as visualized by the loose clustering of the lines associated with the CBSV cluster. Conversely, there is tight clustering of the UCBSV samples indicating more confidence in the relationships within the species.

Bayesian phylogenetic analysis clearly separated CBSV and UCBSV into 2 groups, indicating that they are distinct species (Fig 2). The analyses of the whole genomes and the individual genes were largely congruent with differences in the topologies (Table 4). DensiTree [40], is a visualization tool that displays all trees encountered as the Markov Chains samples the large tree space. The variation in the trees sampled in ExaBayes is shown in Fig 5, and it is apparent there are at least two distinct clades or species present -CBSV and UCBSV. There is more uncertainty in the CBSV relationships as can be seen in the loose clustering of the lines associated with that cluster. Conversely, there is a very tight clustering of the UCBSV sequences, which gives us a higher confidence in the relationships within this species. In addition, an individual with no prior knowledge of the current species delimitations performed these analyses blindly. It took seven hours to run ExaBayes on 384 cores of the Magnus supercomputer and generate one phylogenetic tree using these methods. On a standard laptop this would have taken 609 computing hours or around 25 days on a standard quad-core laptop.

**Table 4:** Comparison of all phylogenetic trees showing and the complete genome tree (NA)-Fig 2 is the reference. (+) Indicates that the clade contains the same species as Fig 2 and occurs in the same location on the tree. (-) Indicates that the clade contains different species and/or is in a different location on the tree when compared to Fig 2.

Genomic region	Clade A	Clade B	Clade C	Clade D	Clade E	Clade F
Whole genome (AA)	+	+	+	+	+	+
Whole genome (NA)	+	+	+	+	+	+
P1 (NA)	+	+	+	+	-	-
P1 (AA)	+	+	+	+	-	-
P3 (NA)	+	+	+	+	+	+
P3 (AA)	+	+	+	-	-	-
6K1 (NA)	+	+	+	-	-	-
6K1 (AA)	-	-	+	-	-	-
CI (NA)	+	+	+	+	+	+
CI (AA)	+	+	+	+	+	+
6K2 (NA)	+	+	+	+	-	-
6K2 (AA)	+	+	+	+	-	-
Nia_Pro (NA)	+	+	+	+	-	-
Nia_Pro (AA)	-	-	-	-	+	-
Nia_VPG (NA)	+	+	+	+	+	+
Nia_VPG (AA)	+	+	+	+	-	-
Nib (NA)	+	+	+	-	-	-
Nib (AA)	+	+	-	-	-	-
HAM-like (NA)	-	-	-	+	+	-
HAM-like (AA)	-	-	-	+	+	-
CP (NA)	+	+	-	-	-	-
CP (AA)	-	-	-	-	-	-

### Species delimitation

CBSV and UCBSV are supported as distinct species as shown in Table 5. There is also further support for three new species in the UCBSV species clade. Clades labeled C, D and E (Fig 2) have significant species delimitation values for P (Randomly Distinct), Clade Support and Rosenberg's P(AB). Clade C contains UCBSVTZ:Tan–23 and UCBSV-UG-MI-B3-FJ039520, Clade D consists of UCBSVTZ:Ser–5 and UCBSVTZ:Ser–6 and Clade E contains UCBSVTZ:MAF–58 and UCBSVTZ:MAF–51. Clade support (PP) is data-driven and is a measure of how strongly the data support the particular clade. In contrast, the P(AB) measure is dependent on the estimated tree topology and on the data only through the estimated tree. More specifically, the null hypothesis for both P(AB) and P(RD) is based on panmixis. As a test for cryptic species identification or species distinctiveness, P(AB) and P(RD) are based on the coalescent [41] and can be applied to genetic data from one locus or whole genomes. P(RD) is defined as the probability of an observed degree of distinctiveness.

**Table 5:** The species delimitation plugin generates: Intra Dist: average pairwise tree distance among members of a predefined clade, Inter Dist: average pairwise tree distance between members of the group of interest and its sister taxa (K2Pdistance), Intra/Inter: The ratio of Intra Dist to Inter Dist, P ID(Liberal): mean probability, with a 95% confidence interval (CI) for a prediction of making a correct identification of an unknown specimen being sister to or within the group of interest, P ID (Strict): mean probability, with a 95% confidence interval (CI) for a prediction of making a correct identification of an unknown specimen being found only in the group of interest [56], Av(MRCA): mean distance between the most recent common ancestor of the species and its members, P(Randomly Distinct): probability that a clade has the observed degree of distinctiveness [38], Clade Support: Bayesian posterior probability (PP), and Rosenberg’s P_AB_: Reciprocal monophyly.

**CBSV versus UCBSV**											
**Species**	**Closest Species**	**Monophyletic?**	**Intra Dist**	**K2P**	**Intra/Inter**	**P ID(Strict)**	**P ID(Liberal)**	**Av(MRCA-tips)**	**P (Randomly Distinct)**	**Clade Support**	**Rosenberg's P(AB)**
CBSV	UCBSV	yes	0.123	1.327	0.09	0.81 (0.67, 0.95)	0.96 (0.85, 1.0)	0.0726	0.05*	1*	3.80E-05*
UCBSV	CBSV	yes	0.12	1.327	0.09	0.95 (0.88, 1.0)	0.99 (0.94, 1.0)	0.1185	0.05*	1*	3.80E-05*
**UCBSV**											
**Species**	**Closest Species**	**Monophyletic?**	**Intra Dist**	**K2P**	**Intra/Inter**	**P ID(Strict)**	**P ID(Liberal)**	**Av(MRCA-tips)**	**P (Randomly Distinct)**	**Clade Support**	**Rosenberg's P(AB)**
C	D	yes	0.102	0.185	0.55	0.31 (0.15, 0.46)	0.64 (0.49, 0.80)	0.0512	0.05*	1*	1.69E-03*
D	E	yes	0.007	0.09	0.08	0.55 (0.40, 0.70)	0.93 (0.78, 1.0)	0.0037	0.05*	1*	2.75E-03*
E	D	yes	0.035	0.09	0.39	0.39 (0.24, 0.54)	0.74 (0.59, 0.90)	0.0177	0.93*	1*	4.94E-03*
F	E	yes	0.049	0.083	0.59	0.40 (0.22, 0.58)	0.67 (0.53, 0.82)	0.037	0.95	0.759	0.01
E	F	yes	0.064	0.083	0.77	0.42 (0.29, 0.54)	0.73 (0.63, 0.84)	0.0409	0.79	0.9209	0.01

* Indicates significant species delimitation.

## Discussion

In the present study, we report the occurrence of 12 new whole genome sequences for the devastating cassava viruses (five new isolates of CBSV and seven UCBSV) from East Africa. We have utilized existing data, NGS technology and Supercomputing to identify new viruses and scrutinize the distribution of CBSV and UCBSV in Tanzania. In doing so, we have found that both viruses are widely distributed in different agro-ecologies (low, medium and high altitude at < or > 1000 m above sea level) in Tanzania. These findings are contrary to previously published reports that CBSV occurrence is limited to low and medium altitude areas below 1000 m above sea level, while UCBSV occurred in the highland areas (>1000 m above sea level.) of East Africa [1, 42]. We found UCBSV isolates in the low land coastal areas in Mafia Island on the Indian Ocean and in the Tanga district where UCBSV isolates occurred either in single or double infection with each other or with CBSV isolates. UCBSV isolates also occurred widely in coastal lowland of Kenya according to Mbanzibwa et al. [8]. Isolates of both CBSV and UCBSV were also found to occur in the highlands in the Lake Zone such as Mara region in single and double infection. In this study, we also report for the first time the occurrence of CBSD due to CBSV in Nyasa district along the shore of the Lake Nyasa in the Ruvuma region in southern Tanzania, where it was previously thought that there was no CBSD [43]. The reasons for the presence of CBSV in Nyasa district, which is located about 1000 km away from the CBSD-endemic areas remains to be investigated, but could be partly due to a combination of factors including planting of susceptible infected sources of cassava materials from CBSD-affected fields in other parts of Tanzania. Until now the spread of CBSD is mainly through use of infected planting materials as reported earlier [44, 45].

In the field, cassava plants affected by CBSD displayed a range of symptoms, depending on the cultivar and agro-ecology. In this study, CBSV and UCBSV were associated with varied foliar symptoms partly because the CBSD resistance and or tolerance levels of the sampled farmers' cassava cultivars could not be established. Our study therefore confirms earlier findings that there is no clear correlation between cassava brown streak virus species and CBSD symptom types and variability [9].

Currently, *Cassava Brown Streak Virus* and *Ugandan Cassava Brown Streak Virus* are the two recognized viral species associated with the devastation of cassava crops in East Africa in recent years [1–3]. The current criterion for distinguishing CBSV from UCBSV is percentage nucleotide similarity (70%) and polyprotein amino acid sequences (74%), which are outlined in an accepted proposal to the International Committee on Taxonomy of Viruses, put forth in 2010 (http://www.ictvonline.org/proposals/2010.001aP.A.v2.Ipomovirus-Sp.pdf). Species delimitation has moved beyond relying on sequence percentage similarity and more robust techniques are needed to distinguish these species. The species delimitation metrics utilized in our study indicates support for these two species but also an additional three species within the UCBSV clade. The new species we have identified are labeled C, D and E (Fig 2) and these putative new species have significant species delimitation values for P (Randomly Distinct), Clade support and Rosenberg's P(AB). Clade C contains UCBSV TZ:Tan–23 and UCBSV-UG-MI-B3-FJ039520, Clade D consists of UCBSV TZ:Ser–5 and UCBSV TZ-Ser–6 and Clade E contains UCBSV TZ:MAF–58 and UCBSV TZ-MAF-51.What are the implications for the identification of the new species? There is a need for extensive biological investigations into these putative species of UCBSV. In addition, a taxonomic revision is necessary and should be completed in accordance with the International Committee on Taxonomy of Viruses [46].

The International Committee on Taxonomy of Viruses (ICTV) has the mandate to name new viruses. One of the essential principles of virus nomenclature aims to avoid or reject the use of names, which might cause error or confusion [47]. One case of 'error', that has caused immense confusion was the naming of one of the two species causing CBSD in East Africa; *Ugandan cassava brown streak virus* (UCBSV). While it is clear that UCBSV is a separate species to CBSV, it is not found exclusively in Uganda, nor did it originate there. Therefore, referring to the species as 'Uganda' has led many to believe that this species has only recently spread or been moved (through use of infected cuttings) to the wider geographical scope it is found in Kenya, Rwanda, Tanzania including mafia and Zanzibar Islands in the Indian Ocean. It would be inconceivable that through recent human effort, UCBSV-infected materials were moved to these islands in the Indian Ocean, which are many miles away from Uganda. Therefore, great care must be taken when naming the clades, groups and species associated with a particular set of sequences and their phylogenetic analysis. It has been common practice to name these according to things such as host, geographic location or country of origin, however this is problematic. It is becoming clear that the use of a numbered, lettered or latinised numeral system may be a useful tool particularly regarding species delimitation and strain differentiation of some plant viruses [33, 48]. This allows for the addition of new groups at a later date, and allows for the fact that there may be changes to makeup of each group depending on the other sequences used in any one analysis. The discussion can then be around the relationship of each clade to each other, and the sequences that are contained within each group rather than whether or not a particular sequence belongs to the “Ugandan” group (e.g. this study). Similar naming systems have been either adopted, or recently proposed in a number of other viruses, particularly the potyviruses [12, 48, 49].

Unbiased species delimitation is a crucial first step in identifying out-breaking viral strains. If traditional species aren’t questioned or evaluated, many new species will go undetected, possibly leading to increased damage due to lack of adequate diagnostics. The use of the Magnus supercomputer made these analyses possible, and without it the 7-hour runtime required to generate one phylogenetic tree would balloon out to approximately 609 hours on a modern laptop or PC. The analyses has allowed us to determine that new diagnostic primers for the individual species should be redesigned for the CI region of the genome–Table 4 shows it to be most stable in terms of the individual gene trees, for both nucleotide and amino acid. No other gene region displays this stability. This is an interesting finding because most diagnostic primers are based on the CP region of the genome [8].

As in previous studies, we found no evidence of recombination between CBSV and UCBSV, although there is evidence for homologous recombination [50]. As more and more isolates of the two viruses have their full genomes sequenced, it will be of great interest to conduct further recombination analysis, and further species delimitation calculations. It is not surprising to have identified homologous recombination here, and not unreasonable to expect that if it hasn’t already occurred then they may be opportunities for recombination between the virus species given that Potyviruses are well known for their readiness to undergo recombination [24, 33, 51–53].

A way forward in identifying plant viruses affecting cassava should include the rigor that has been established in the current study, NGS technologies coupled with proper phylogenetics and species delimitation metrics are necessary for identifying cassava plant viruses. The data presented shows there is a need to reevaluate the current diagnostic primers (coat protein and 3’ UTR) for CBSV/UCBSV as they are not detecting the diversity, this is key to ensuring clean seeds and cuttings are moved throughout the region. In addition, it will be crucial as more viruses are discovered to map the transmission efficiency of these viruses with the new species of the vector, *Bemisia tabaci*, that are currently being discovered [54, 55]. Integration of vector virus interactions will be the key to increasing cassava productivity.
