# Correction: Analyses of Twelve New Whole Genome Sequences of Cassava Brown Streak Viruses and Ugandan Cassava Brown Streak Viruses from East Africa: Diversity, Supercomputing and Evidence for Further Speciation

**DOI:** 10.1371/journal.pone.0141939

**Published:** 2015-10-28

**Authors:** Joseph Ndunguru, Peter Sseruwagi, Fred Tairo, Francesca Stomeo, Solomon Maina, Appolinaire Djikeng, Monica Kehoe, Laura M. Boykin

The six author’s name is incorrectly spelled. The correct name is: Appolinaire Djikeng. The correct citation is: Ndunguru J, Sseruwagi P, Tairo F, Stomeo F, Maina S, Djikeng A, et al. (2015) Analyses of Twelve New Whole Genome Sequences of Cassava Brown Streak Viruses and Ugandan Cassava Brown Streak Viruses from East Africa: Diversity, Supercomputing and Evidence for Further Speciation. PLoS ONE 10(10): e0139321. doi:10.1371/journal.pone.0139321

## References

[1] Ndunguru J, Sseruwagi P, Tairo F, Stomeo F, Maina S, Djinkeng A, et al (2015) Analyses of Twelve New Whole Genome Sequences of Cassava Brown Streak Viruses and Ugandan Cassava Brown Streak Viruses from East Africa: Diversity, Supercomputing and Evidence for Further Speciation. PLoS ONE 10(10): e0139321 doi:10.1371/journal.pone.0139321 26439260 10.1371/journal.pone.0139321 PMC4595453

